# Association between periodontitis and temporomandibular joint disorders

**DOI:** 10.1186/s13075-023-03129-0

**Published:** 2023-08-08

**Authors:** Shaotai Wang, Huan Jiang, Huichuan Qi, Danfeng Luo, Tianyuan Qiu, Min Hu

**Affiliations:** 1 Department of Orthodontics, Hospital of Stomatology, Jilin University, Changchun, 130021 China; 2 Jilin Provincial Key Laboratory of Tooth Development and Bone Remodeling, Changchun, 130021 China

**Keywords:** Periodontitis, Temporomandibular joint disorders, Mendelian randomization

## Abstract

**Background:**

Periodontitis (PD) may affect temporomandibular joint disorders (TMD) and TMD may influence PD in previous observational studies. Nevertheless, these studies were prone to confounders and reverse causation, leading to incorrect conclusions about causality and direction of association. This research investigates the associations between PD and TMD employing bidirectional two-sample Mendelian randomization (MR) analysis.

**Methods:**

Single-nucleotide polymorphisms (SNPs) related to PD (*p* < 5 × 10^−6^) were selected from a genome-wide association study (GWAS) from the Gene-Lifestyle Interaction in the Dental Endpoints (GLIDE) consortium, and related these to SNPs from FinnGen and UK Biobank (UKB) consortia, and vice versa. We implemented the standard inverse variance weighted (IVW), weighted median (WM), MR-Egger regression, and MR-PRESSO methods to estimate the potential causality between PD and TMD. Sensitive tests were conducted using robust MR methods. Results from FinnGen and UKB were combined using the fixed model.

**Results:**

PD did not appear to causally affect TMD. Additionally, the reverse MR analysis did not reveal a significant causal effect of TMD on PD. The results of other MR methods were similar to those of the IVW method. Sensitivity analyses addressed no potential pleiotropy in MR estimations. Results from the meta-analysis were consistent with the above-mentioned consequences.

**Conclusion:**

This research does not support a causal relationship between PD and TMD. PD does not appear to worsen TMD directly, and vice versa.

**Supplementary Information:**

The online version contains supplementary material available at 10.1186/s13075-023-03129-0.

## Introduction

Periodontitis (PD) is the six-most prevalent disease around the world and the first cause for tooth loss among adults [1]. The host’s innate immune system mounts a defense response once infected with oral periodontal pathogenic bacteria [2]. Inflammatory risk factors for periodontal diseases are similar to those for systemic and chronic inflammatory diseases. PD and other diseases may have causal associations. Experimental studies showing biologically plausible and clinically consistent mechanisms by which PD might initiate or aggravate comorbid conditions further support the causal link between PD and comorbidities [3].

In clinical cases, patients with PD often suffer from temporomandibular joint disorders (TMD). TMD refers to several diverse neuromuscular and musculoskeletal conditions including the temporomandibular joint complex as well as surrounding muscular and skeletal structures. Jaw pain or dysfunction, headache, earache, and facial pain are common symptoms. TMD is the result of a combination of biological, emotional, cognitive, environmental, and social factors [4]. The diagnosis of TMD will be based on a detailed medical examination. Radiology examinations might include panoramic X-rays, CT scans, and MRIs. TMD can be aggravated by incorrect disc positioning, cartilage damage, and bruxism [5]. Previous observational studies have shown that the space vertical to the condyles and the distance between the outer and inner poles of the condyle might change over time [6].

There is a high prevalence of PD and TMD among adults worldwide. Unilateral mastication due to PD could induce pain and structural changes in the temporomandibular joint [7]. In addition to clinical observational studies, two symptoms often occur together in many diseases, such as rheumatic diseases [8, 9] and multiple sclerosis [10]. If PD and TMD are causally related, oral health will be threatened, and these diseases are challenging to treat. In spite of the fact that such previous findings pointed towards PD as a potential risk factor for TMD or TMD as a risk factor for PD, several problems remained. As a result of the reverse causation and existence of unknown or unmeasured confounders, the association between exposure and outcome would be biased in observational studies.

Genetic variants can function as instrumental variables (IVs) in Mendelian randomization (MR) analyses to assess the causality of exposure on outcome. Several gene variants were found to be associated with PD in a moderate to strong way [11]. A number of SNPs also exert a role in TMD susceptibility [12–14]. An IV must satisfy the following fundamental conditions: (1) the variant is robustly related to exposure; (2) the variant must not be related to outcome through confounders; (3) there is no direct correlation between the variant and the outcome, but perhaps indirect correlation through exposure [15]. Here, we analyzed PD data from the Gene-Lifestyle Interaction in the Dental Endpoints (GLIDE) consortium [16]. TMD data were derived from FinnGen [17] and UK Biobank (UKB) consortia [18]. The ultimate aim of this MR research is to clarify the potential causal association between PD and TMD and to corroborate previous studies.

## Methods

**Fig. 1:**
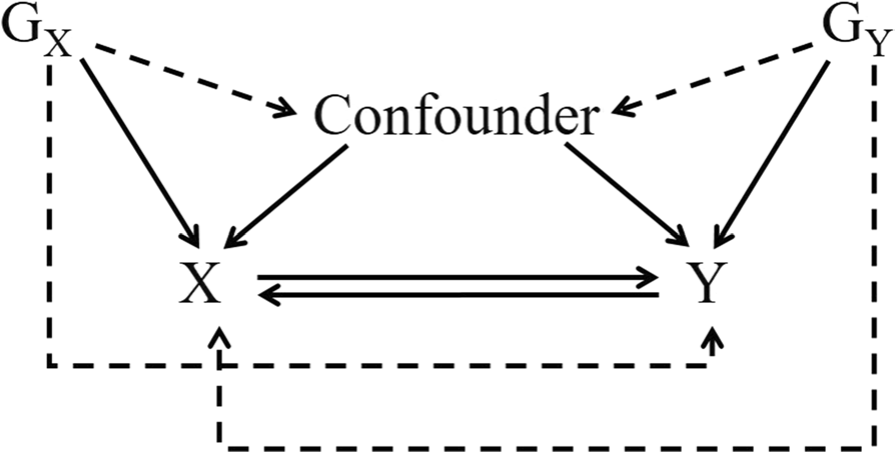
Directed graph of bidirectional two-sample MR analysis evaluating the potential causality between X and Y. Genetic variants G_X_ can be used to estimate the causal effect of exposure X on outcome Y. Genetic variants G_Y_ can be used to estimate the causal effect of outcome Y on exposure X. The solid and dashed lines denote causal and noncausal effects, respectively. An arrow from X to Y indicates a causal effect of X on Y directly

Bidirectional MR (Fig. 1) assesses both the effect of the exposure on the outcome and the effect of the outcome on the exposure. Our study examined possible causal associations between PD and TMD using bidirectional two-sample MR analysis based on statistics from genome-wide association studies (GWAS).

### Data source

**Fig. 2:**
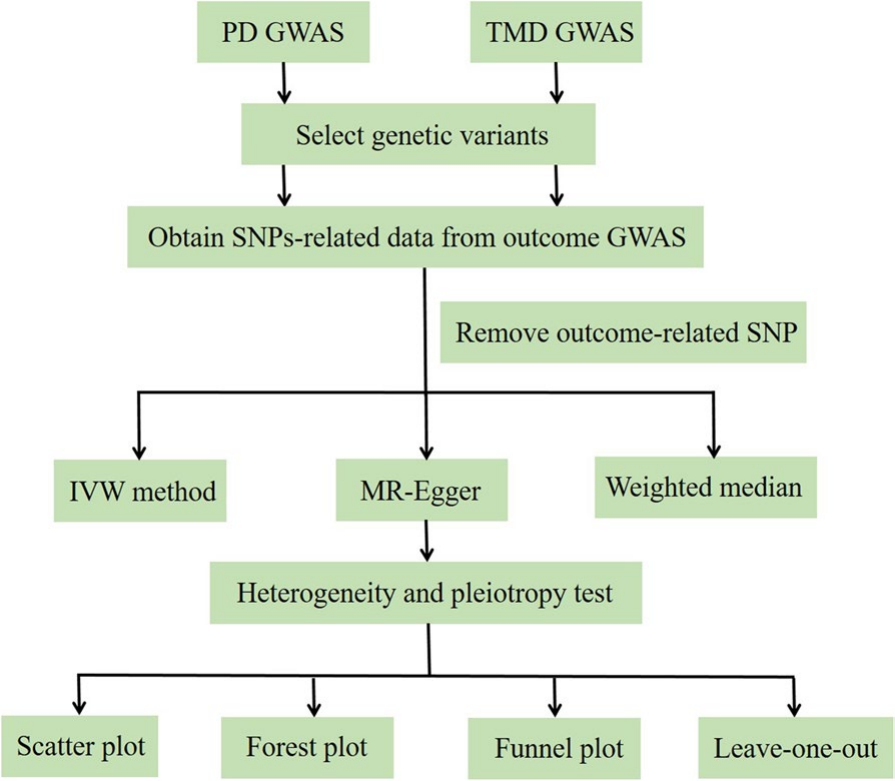
Workflow of the MR study revealing the causality between PD and TMD

Figure 2 presents an overview of this study. GWAS data for PD were derived from the GLIDE consortium, including 17,353 cases and 28,210 controls [19]. After excluding the Hispanic/Latino people, there were 12,289 European cases and 22,326 European controls. TMD data were derived from FinnGen consortium in discovery stage, including 4273 cases and 177,661 controls [17]. In the UKB consortium GWAS, there were 217 European ancestry cases, together with 456,131 European ancestry controls [18].

For the first MR assumption, we selected IVs that were robustly associated with PD and TMD with *p* value less than 5 × 10^−8^, respectively. To obtain more SNPs, we broadened the threshold to 5 × 10^−6^. We then performed a procedure to exclude SNPs in strong linkage disequilibrium (LD) by using *R*^2^ < 0.001 and a window size of 10 mb. To fulfill the third assumption, the *p* value of the outcome is larger than 5 × 10^−8^. Stress, cold stimulation, occlusal interference, and other TMD risk factors were examined to determine whether pleiotropic ways affected MR estimations. Rs2976950 was removed from the selected SNPs. We also found several common risk factors for PD, such as smoking, diabetes, etc. As a substitute for associated SNP not included in outcome data, we used proxy SNP (LD R^2^ > 0.8). Finally, the palindromic and incompatible SNPs were excluded by harmonizing the exposure and outcome data. As a result, 6 SNPs (Supplementary Table 1) were used to assess the potential causal effect of PD on TMD in the discovery stage, 7 SNPs (Supplementary Table 2) were used in the validation stage, and 12 SNPs (Supplementary Table 3) were used to evaluate the causal effect in the opposite direction.

### Statistical analyses

In this study, four MR methods were implemented in order to estimate the possible causal effect between PD and TMD. Inverse variance weighted (IVW) method was applied for the principal analysis. IVW approach is the most efficient MR method, biased if the average pleiotropic effect differs from zero [20]. As complements to the IVW analysis, other MR analyses were performed, such as the weighted median (WM), the MR-Egger regression, and the MR-PRESSO. WM is robust to outliers and sensitive to genetic variant additions or deletions [21]. MR-Egger is robust to pleiotropy under the InSIDE assumption, sensitive to outliers, and sensitive to violations of the InSIDE assumption [22].

To verify the reliability of the results, we implemented sensitive analyses, such as Cochran’s *Q* test, MR-Egger intercept test, and MR-PRESSO global test. Results of Cochran’s *Q* test (*p* value < 0.05) indicate heterogeneity. The MR-Egger intercept test can be used to assess horizontal pleiotropy. MR-PRESSO can remove outliers and is efficient with valid IVs [23]. Using a leave-one-out sensitivity analysis, we were able to determine whether causal effects were influenced by a single SNP by removing each exposure-associated SNP individually. A fixed-effect model was used to estimate the effect of combined results derived from FinnGen and UKB consortia.

Statistical significance was set at 0.05 for all *p* values (two-sided). Using odds ratios (OR) with 95% confidence intervals (CI), the MR analysis results provided an estimation of the outcome risk associated with each standard deviation (SD) increase in exposure, either PD or TMD. In order to eliminate SNPs associated with potential risk confounder factors, we checked the SNPs selected by the rules in PhenoScanner (http://www.phenoscanner.medschl.cam.ac.uk), a website with comprehensive information about the association between genotype and phenotype. Select according to the default criteria (*p* < 1 × 10^−5^, *r*^2^ > 0.8). Proxy SNPs are found by using a website tool (https://snipa.helmholtz-muenchen.de/snipa3/). TwoSampleMR (version 0.5.6) and MRPRESSO (version 1.0) packages were installed in R (version 4.2.0) for MR analyses. Meta-analyses were conducted in StataMP (version 17).

## Results

### MR estimates for PD on TMD in the discovery stage

There was no significant evidence of PD being causally linked to TMD by selecting 6 PD-related SNPs. The results of the IVW (OR = 0.977, 95% CI = 0.886 ~ 1.077, *p* = 0.640) method showed that PD was not causally related to TMD. MR-Egger (OR = 0.976, 95% CI = 0.866 ~ 1.101, *p* = 0.713) and WM (OR = 0.959, 95% CI = 0.849 ~ 1.083, *p* = 0.500) results showed a consistent but not significant direction.

Sensitivity analyses were implemented to test the reliability of the results above, such as Cochran’s *Q* test, MR-Egger intercept test, and MR-PRESSO global test. There was no heterogeneity between PD and TMD in the *Q* test (IVW *Q* = 2.810, *p* = 0.832). Also, the MR-PRESSO global test demonstrated a similar result (*p* = 0.724), and no outliers were eliminated. According to the pleiotropy test, there was no significant intercept (Egger intercept = 0.0003, SE = 0.0193, *p* = 0.988), indicating no horizontal pleiotropy.

### MR estimates for PD on TMD in the validation stage

We replicated the MR analysis in UKB data set. There was no significant evidence of PD being causally linked to TMD by selecting 7 PD-related SNPs. The results of the IVW (OR = 1.200, 95% CI = 0.729 ~ 1.975, *p* = 0.474), MR-Egger (OR = 1.052, 95% CI = 0.526 ~ 2.103, *p* = 0.892), and WM (OR = 1.172, 95% CI = 0.631 ~ 2.175,* p* = 0.615) showed a consistent but not significant direction.

No heterogeneity was observed between PD and TMD in the *Q* test (IVW *Q* = 3.371, p = 0.761). Also, the MR-PRESSO global test demonstrated a similar result (*p* = 0.830), and no outliers need to be eliminated. From the pleiotropy test, there was no significant intercept (Egger intercept = 0.0656, SE = 0.1223, *p* = 0.615), indicating no horizontal pleiotropy.

### Combining results of the discovery and validation stage

**Fig. 3:**
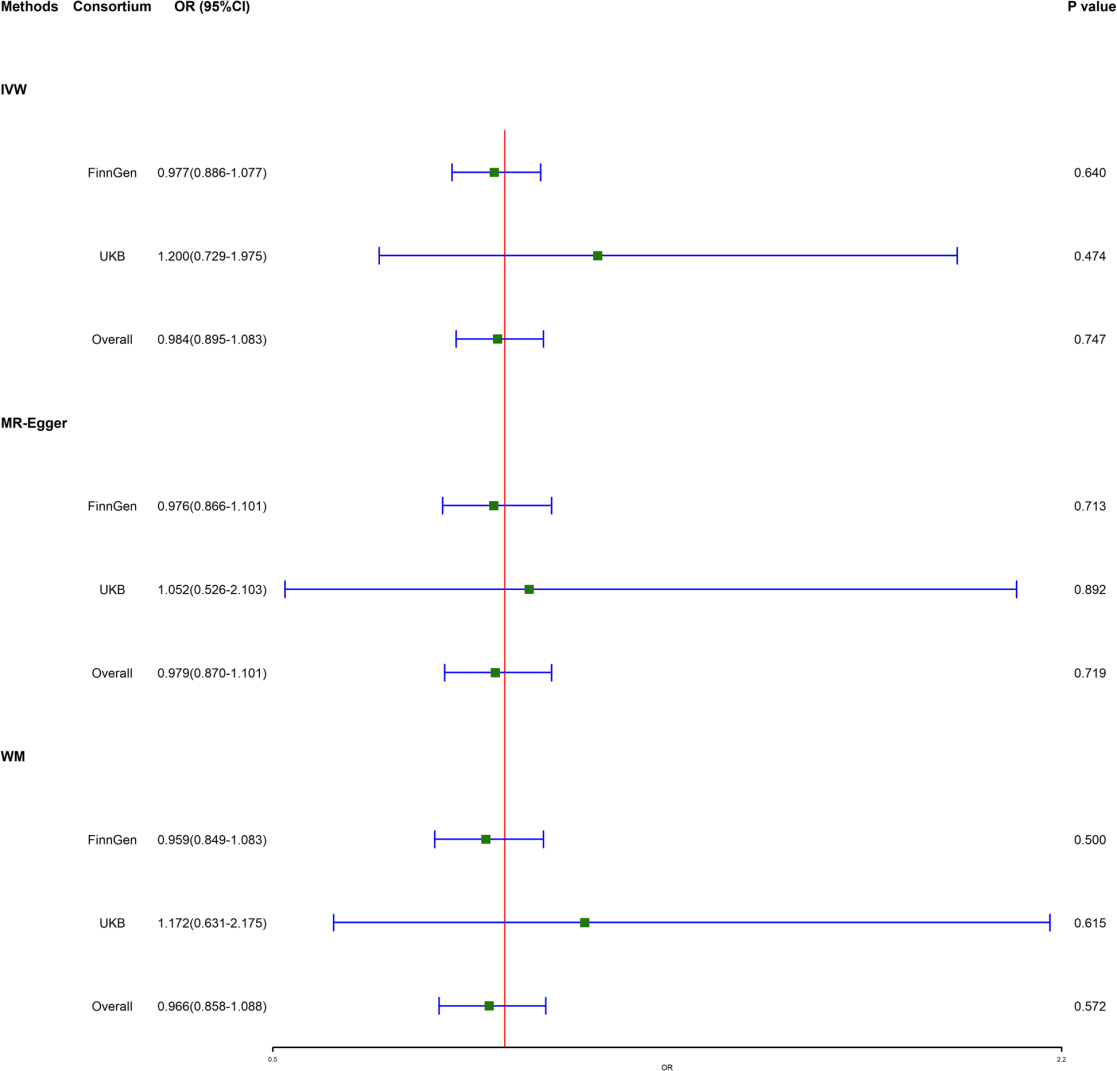
Estimated causal effects between PD and TMD using IVW, MR-Egger, and WM method. OR, odds ratio

Figure 3 summarized the MR estimation results from FinnGen and UKB consortium. The results from these two consortia could be deemed consistent. The meta-analysis of MR results also confirmed TMD could not be causally affected by PD.

### MR estimates for TMD on PD

There was no significant evidence of TMD being causally linked to PD by selecting 12 TMD-related SNPs. The results of IVW (OR = 0.957, 95% CI = 0.844 ~ 1.085, *p* = 0.490) showed that TMD was not causally associated with PD. MR-Egger (OR = 0.910, 95% CI = 0.666 ~ 1.244, *p* = 0.567) and WM (OR = 0.952, 95% CI = 0.808 ~ 1.122, *p* = 0.561) showed consistent conclusions.

There was no heterogeneity between TMD and PD in the *Q* test (IVW *Q* = 13.842, *p* = 0.242). The MR-PRESSO global test also presented a similar result (*p* = 0.215), and no outliers needed to be removed. Nonsignificant intercept existed in the pleiotropy test (Egger intercept = 0.0084, SE = 0.0241, *p* = 0.736), indicating that horizontal pleiotropy might not appear to affect the association between TMD and PD. From the leave-one-out analysis, no single SNP violated the overall effect of TMD on PD. The scatter plot, forest plot, funnel plot, and leave-one-out analysis plot of SNPs associated with PD and TMD were shown in Supplementary Figures.

## Discussion

In this research, we implemented a bidirectional two-sample MR analysis based on GWAS data from the GLIDE, FinnGen, and UKB consortia. This MR analysis confirmed that no significant evidence supports the causal association between PD and TMD.

In clinical observations, TMD always accompanies patients with PD. When PD progresses, inflammation-induced discomfort may cause an abnormal occlusal relationship or even occlusal trauma, leading to back-shift of the condyle, midline deviation, and TMD [24]. Until now, causal relations between PD and TMD remain unclear. The reverse effect still exists in clinical observational studies. In addition, previous observations were difficult to exclude confounding risk factors, while using MR analysis may avoid these problems. We used two sets of genetic instruments to represent PD and TMD in the MR analysis.

Though the results showed no causality between PD and TMD, PD might affect the progression of TMD, and it also might be true in the opposite direction. In advanced cases, PD results in irreversible destruction of the periodontium and tooth loss [25], leading to occlusal dysfunction. Based on a retrospective study of 4204 randomly selected patients, significant associations were found between missing teeth and difficulty chewing [26], which damaged the joints. An increase in mechanical overload or microtrauma might lead to TMD in the presence of missing teeth. Furthermore, these circumstances are implicated in intricate biological processes, including the activation of the inflammation and immune system, as well as the extracellular matrix components degradation [27]. Researchers have identified that helper T cells play a pivotal role in numerous autoimmune diseases, which are conventionally associated with periodontal bone loss. PD inflammation might be aggravated by Th17-released molecules, which facilitate the pro-inflammation cascades [28]. Cytokines related to the Th1/Th17/Th22 axis of immune-inflammatory response were involved in temporomandibular joint osteoarthritis [29]. Compared with healthy tissues, the chronic PD sample had an increased expression of protease-activated receptor (PAR)-2 [30]. A neurogenic mechanism involving natural killer 1 receptors activates PAR-2 in the temporomandibular joint, causing inflammation [31]. Periodontal inflammation is triggered and perpetuated by signaling molecules belonging to the interleukin (IL)-1 family [32], and IL-1 receptor 1 was associated with anterior disc displacement with reduction (ADDwR) and anterior disc displacement without reduction (ADDwoR) in TMD discs of humans [33].

However, several limitations should be considered in our study. Firstly, all the GWAS data came from European participants. This method of data processing was capable of maintaining consistency between the sources of data for exposures and outcomes. In spite of this, the results of the MR analysis should be tested in other populations in the future. Secondly, participants in the exposure and outcome studies might have overlapped. Since we used GWAS data from multiple consortia, the impact of overlap was reduced. Thirdly, SNPs selected as IVs were not robustly associated with PD or TMD (*p* value < 5 × 10^−6^), which might influence statistical power to some extent. In this research, we added the number of participants to increase the statistical power.

## Conclusion

The aim of this research is to investigate the potential causality between PD and TMD using a bidirectional two-sample MR method. Our study results find no significant evidence to support the causal effect of PD on TMD, neither does TMD on PD.

### Supplementary Information

**Additional file 1:** **Supplementary Table 1.** Detailed information on IVs after harmonizing the exposure (PD) and outcome (TMD) data in discovery stage. **Supplementary Table 2.** Detailed information on IVs after harmonizing the exposure (PD) and outcome (TMD) data in validation stage. **Supplementary Table 3.** Detailed information on IVs after harmonizing the exposure (TMD) and outcome (PD) data. **Supplementary Figure 1.** Scatter plot of SNPs associated with PD and TMD. **Supplementary Figure 2.** Forest plot of SNPs associated with PD and TMD. **Supplementary Figure 3.** Funnel plot of SNPs associated with PD and TMD. **Supplementary Figure 4.** Leave-one-out analysis plot of SNPs associated with PD and TMD.

## Data Availability

All data used in the current study are publicly available GWAS summary data.
