# Correction: Association between periodontitis and temporomandibular joint disorders

**DOI:** 10.1186/s13075-023-03192-7

**Published:** 2023-10-21

**Authors:** Shaotai Wang, Huan Jiang, Huichuan Qi, Danfeng Luo, Tianyuan Qiu, Min Hu

**Affiliations:** 1 Department of Orthodontics, Hospital of Stomatology, Jilin University, Changchun, 130021 China; 2 Jilin Provincial Key Laboratory of Tooth Development and Bone Remodeling, Changchun, 130021 China

**Correction: Arthritis Res Ther 25, 143 (2023)**

**https://doi.org/10.1186/s13075-023-03129-0**

Following publication of the original article [1], the authors reported an error under heading Introduction.

The sentence currently reads:

17 studies were included (13 cross-sectional and 4 cohorts), indicating that multiple sclerosis patients are more likely to suffer from periodontal diseases and TMD [7]. TMD and periodontal disease might be the first symptoms of several rheumatic diseases [8].

The sentence should read:

Unilateral mastication due to PD could induce pain and structural changes in the temporomandibular joint [7]. In addition to clinical observational studies, two symptoms often occur together in many diseases, such as rheumatic diseases [8, 9] and multiple sclerosis [10].

Updated references:

7. Jeon HM, Ahn YW, Jeong SH, Ok SM, Choi J, Lee JY, Joo JY, Kwon EY: Pattern analysis of patients with temporomandibular disorders resulting from unilateral mastication due to chronic periodontitis. *J Periodontal Implant Sci* 2017, 47(4):211-218.

8. Gualtierotti R, Marzano AV, Spadari F, Cugno M: Main Oral Manifestations in Immune-Mediated and Inflammatory Rheumatic Diseases. *Journal of clinical medicine* 2018, 8(1).

9. González-Chávez SA, Pacheco-Tena C, Campos Torres RM, Quiñonez-Flores CM, Reyes-Cordero G, Caraveo Frescas TJ: Temporomandibular and Odontological Abnormalities in Patients with Rheumatoid Arthritis. *Reumatol Clin (Engl Ed)* 2020, 16(4):262-271.

10. Al-Ansari A: Is there an association between multiple sclerosis and oral health? *Evidence-based dentistry* 2021, 22(1):44-45.

The original article [1] has been updated.
