# Supplementary material for: Association between periodontitis and temporomandibular joint disorders
Source: Arthritis Res Ther. 2023 Aug 8;25:143. doi: 10.1186/s13075-023-03129-0 (PMC10408055; doi:10.1186/s13075-023-03129-0)
Supplement: Supplementary file 1 — Additional file 1: Supplementary Table 1. Detailed information on IVs after harmonizing the exposure (PD) and outcome (TMD) data in discovery stage. Supplementary Table 2. Detailed information on IVs after harmonizing the exposure (PD) and outcome (TMD) data in validation stage. Supplementary Table 3. Detailed information on IVs after harmonizing the exposure (TMD) and outcome (PD) data. Supplementary Figure 1. Scatter plot of SNPs associated with PD and TMD. Supplementary Figure 2. Forest plot of SNPs associated with PD and TMD. Supplementary Figure 3. Funnel plot of SNPs associated with PD and TMD. Supplementary Figure 4. Leave-one-out analysis plot of SNPs associated with PD and TMD. [file 13075_2023_3129_MOESM1_ESM.docx]

**Appendix**

Shaotai Wang^1,2^, Huan Jiang^1,2**^, Huichuan Qi^1,2^, Danfeng Luo^1,2^, Tianyuan Qiu^1,2^, Min Hu^1,2*^

*^1^Department of Orthodontics, Hospital of Stomatology, Jilin University, Changchun 130021, China*

*^2^Jilin Provincial Key Laboratory of Tooth Development and Bone Remodeling, Changchun 130021, China*

**Supplementary Table 1** Detailed information on IVs after harmonizing the exposure (PD) and outcome (TMD) data in discovery stage

| SNP | EA | OA | PD | | |  | TMD | | |
| --- | --- | --- | --- | --- | --- | --- | --- | --- | --- |
|  |  |  | beta | se | p value |  | beta | se | p value |
| rs13005050 | T | C | -0.1432 | 0.031 | 3.76E-06 |  | 0.00460575 | 0.032644 | 0.887799 |
| rs138868497 | T | C | 1.6387 | 0.3324 | 8.20E-07 |  | -0.0694323 | 0.0971572 | 0.474832 |
| rs151226594 | T | G | -0.3671 | 0.0768 | 1.75E-06 |  | 0.0650104 | 0.0769802 | 0.398386 |
| rs4956201 | A | C | -0.2406 | 0.0474 | 3.89E-07 |  | -0.0543046 | 0.0577281 | 0.346861 |
| rs6816769 | T | C | -0.1348 | 0.0294 | 4.57E-06 |  | 0.00618585 | 0.0380482 | 0.87085 |
| rs73155039 | A | G | 0.8316 | 0.1757 | 2.22E-06 |  | 0.1297 | 0.145554 | 0.372887 |

After selecting SNPs by p value and clumping the data, there were eight SNPs left. Rs140388808 was used as a proxy SNP of rs78422482. To eliminate the influence of the confounder, we deleted rs2976950. Then, we removed the rs140388808 for incompatible alleles. There were 6 SNPs left. EA: effect allele; OA: other allele.

**Supplementary Table 2** Detailed information on IVs after harmonizing the exposure (PD) and outcome (TMD) data in validation stage

| SNP | EA | OA | PD | | |  | TMD | | |
| --- | --- | --- | --- | --- | --- | --- | --- | --- | --- |
|  |  |  | beta | se | p value |  | beta | se | p value |
| rs13005050 | T | C | -0.1432 | 0.031 | 3.76E-06 |  | -0.0348 | 0.159622 | 0.827433 |
| rs138868497 | T | C | 1.6387 | 0.3324 | 8.20E-07 |  | 0.271451 | 0.607078 | 0.654771 |
| rs151226594 | T | G | -0.3671 | 0.0768 | 1.75E-06 |  | 0.387642 | 0.397102 | 0.328977 |
| rs4956201 | A | C | -0.2406 | 0.0474 | 3.89E-07 |  | -0.04582 | 0.198643 | 0.817573 |
| rs6816769 | T | C | -0.1348 | 0.0294 | 4.57E-06 |  | -0.09062 | 0.165199 | 0.58332 |
| rs73155039 | A | G | 0.8316 | 0.1757 | 2.22E-06 |  | 0.050899 | 0.439734 | 0.907851 |
| rs78422482 | A | G | 0.2425 | 0.051 | 2.02E-06 |  | 0.40967 | 0.269211 | 0.128073 |

After selecting SNPs by p value and clumping the data, there were eight SNPs left. To eliminate the influence of the confounder, we deleted rs2976950. There were 7 SNPs left. EA: effect allele; OA: other allele.

**Supplementary Table 3** Detailed information on IVs after harmonizing the exposure (TMD) and outcome (PD) data

| SNP | EA | OA | TMD | | |  | PD | | |
| --- | --- | --- | --- | --- | --- | --- | --- | --- | --- |
|  |  |  | beta | se | p value |  | beta | se | p value |
| rs10882591 | T | C | -0.197625 | 0.0402223 | 8.95E-07 |  | -0.0615 | 0.033 | 0.06235 |
| rs17017794 | C | T | 0.195693 | 0.0420266 | 3.22E-06 |  | -0.0292 | 0.0493 | 0.5533 |
| rs184920366 | A | G | 0.337796 | 0.0739245 | 4.89E-06 |  | -0.2729 | 0.2018 | 0.1764 |
| rs28497436 | C | T | 0.105372 | 0.0228169 | 3.87E-06 |  | -0.0263 | 0.0189 | 0.1634 |
| rs3024577 | G | A | -0.103926 | 0.0226321 | 4.39E-06 |  | -0.0028 | 0.0176 | 0.8729 |
| rs34459436 | A | G | 0.108601 | 0.0237771 | 4.94E-06 |  | 0.0061 | 0.0462 | 0.8951 |
| rs4739605 | T | C | 0.825826 | 0.177928 | 3.46E-06 |  | 0.1176 | 0.1638 | 0.4727 |
| rs72695283 | G | T | 0.119877 | 0.0259341 | 3.79E-06 |  | 0.0145 | 0.0215 | 0.4987 |
| rs73082331 | A | G | -0.139848 | 0.0306299 | 4.98E-06 |  | 0.0135 | 0.0302 | 0.6545 |
| rs73123505 | A | G | -0.145101 | 0.0293567 | 7.71E-07 |  | 0.0067 | 0.0271 | 0.8048 |
| rs78441633 | T | C | 0.225434 | 0.0492612 | 4.73E-06 |  | -0.0163 | 0.0409 | 0.6899 |
| rs78674086 | G | A | -0.386927 | 0.084655 | 4.86E-06 |  | 0.1364 | 0.0586 | 0.0199 |

After selecting SNPs by p value and clumping the data, there were 13 SNPs left. Then, we removed the rs73220964 for being palindromic with intermediate allele frequencies. There are 12 SNPs left.

**Supplementary Figure 1** Scatter plot of SNPs associated with PD and TMD

1. MR estimates for PD on TMD in discovery stage
2. MR estimates for PD on TMD in validation stage
3. MR estimates for TMD on PD


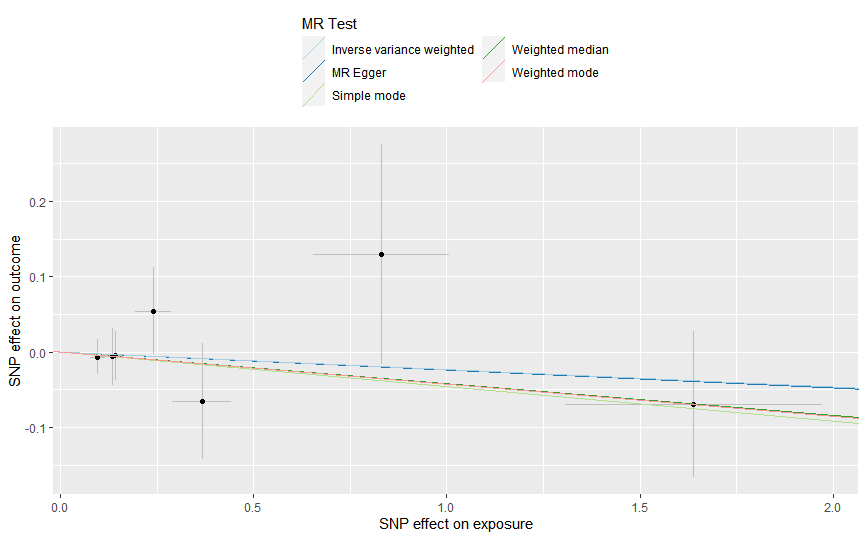


(a)


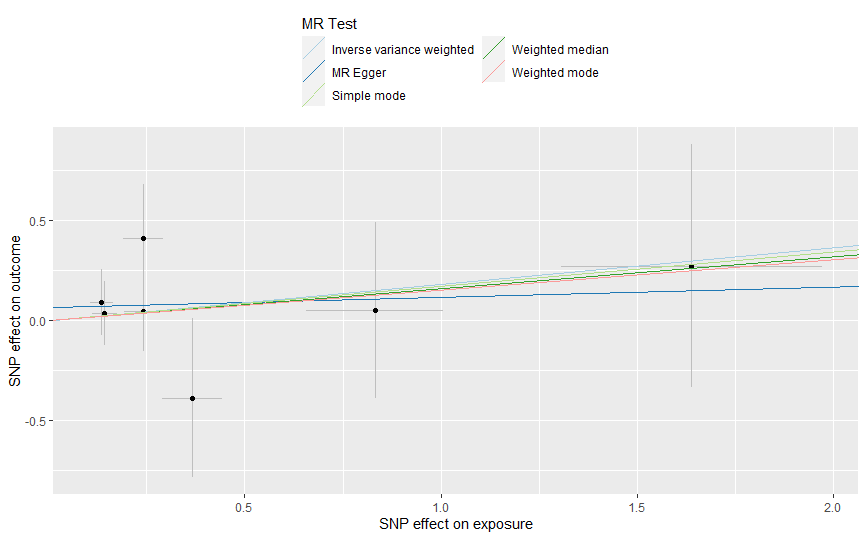


(b)


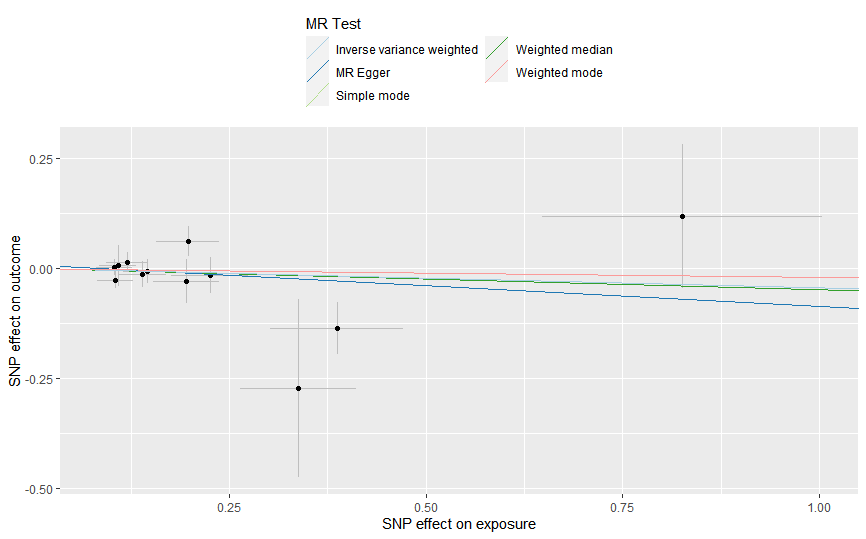


(c)

**Supplementary Figure 2** Forest plot of SNPs associated with PD and TMD

1. MR estimates for PD on TMD in discovery stage
2. MR estimates for PD on TMD in validation stage
3. MR estimates for TMD on PD


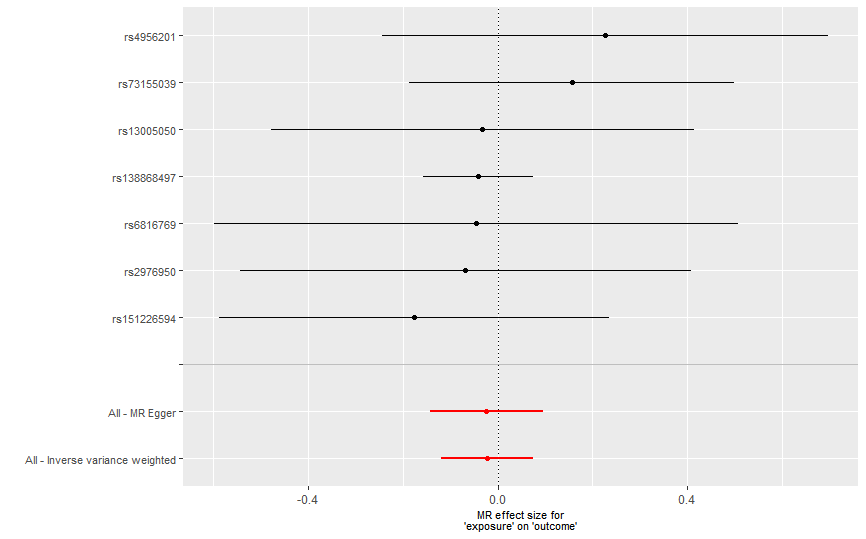


(a)


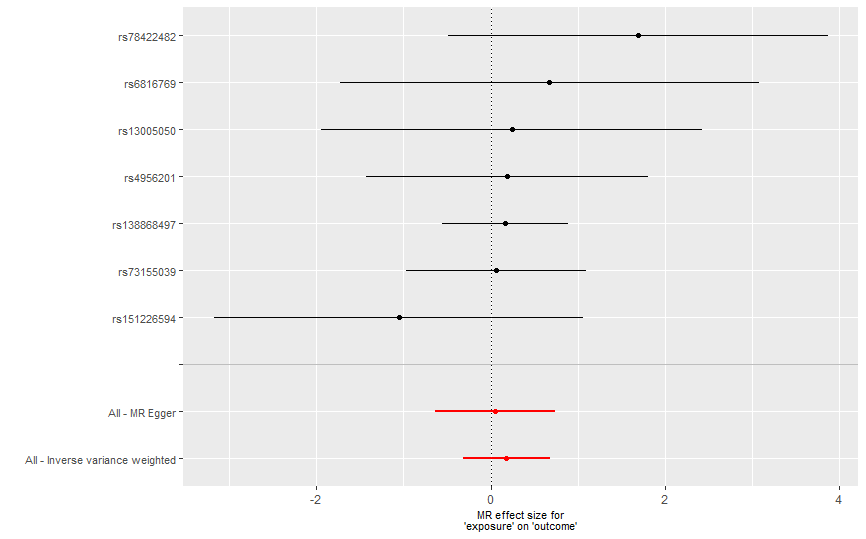


(b)


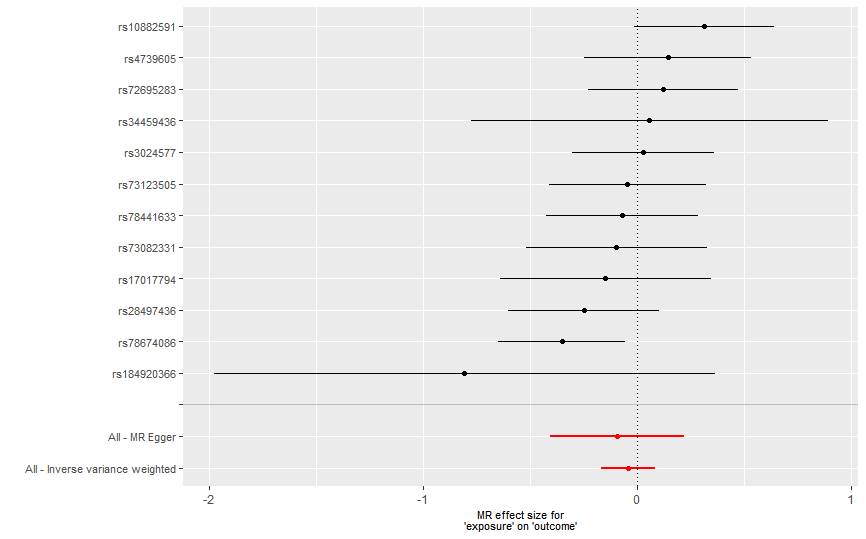


(c)

**Supplementary Figure 3** Funnel plot of SNPs associated with PD and TMD

1. MR estimates for PD on TMD in discovery stage
2. MR estimates for PD on TMD in validation stage
3. MR estimates for TMD on PD


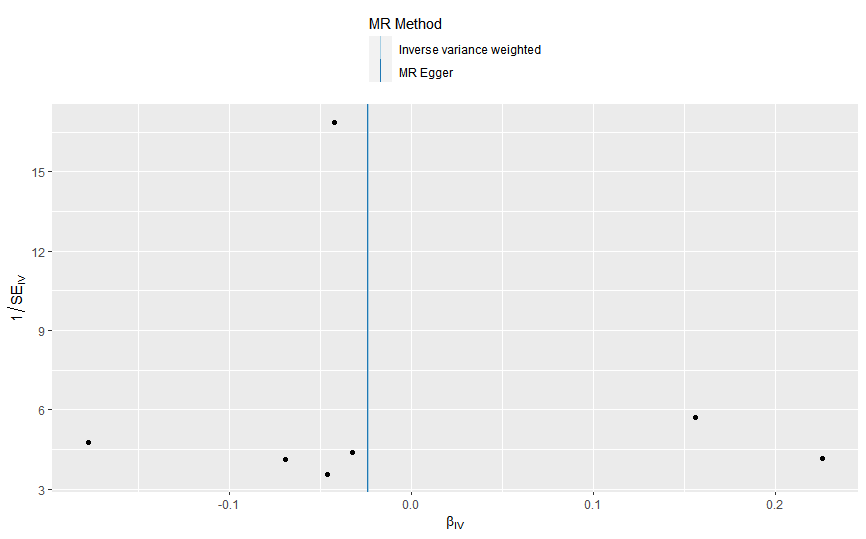


(a)


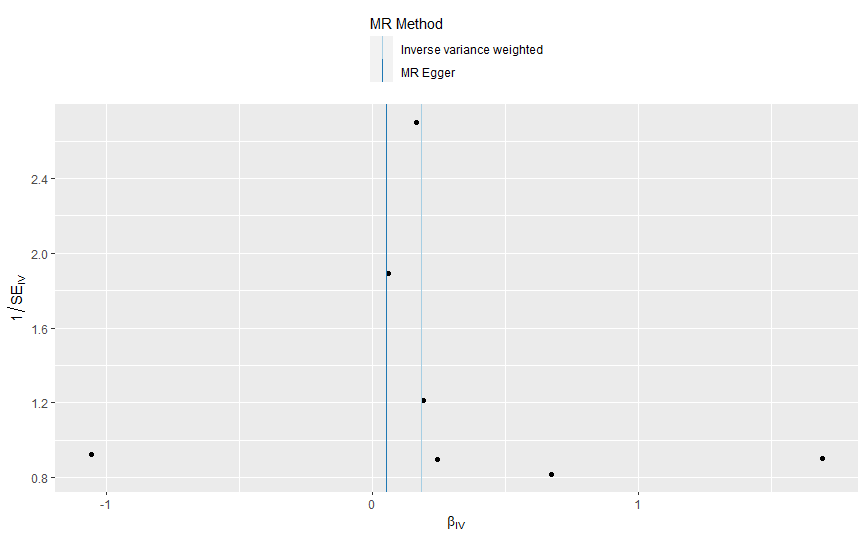


(b)


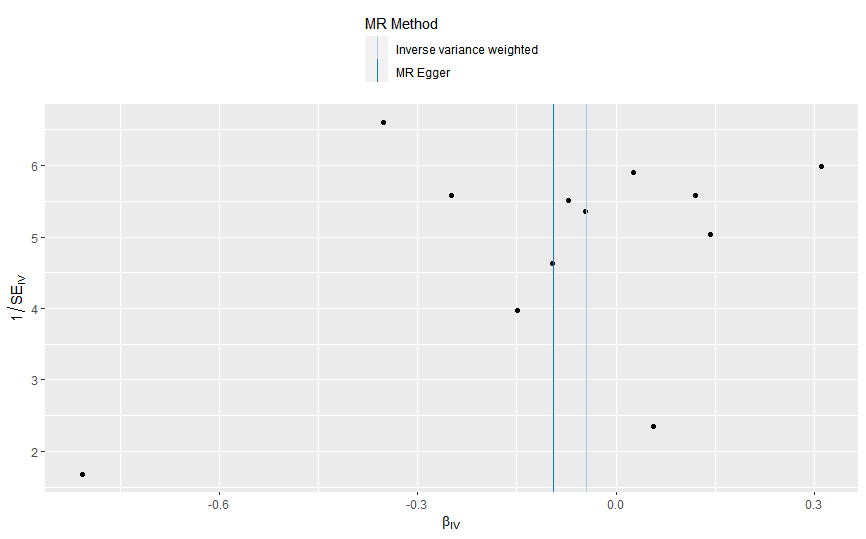


(c)

**Supplementary Figure 4** Leave-one-out analysis plot of SNPs associated with PD and TMD

1. MR estimates for PD on TMD in discovery stage
2. MR estimates for PD on TMD in validation stage
3. MR estimates for TMD on PD


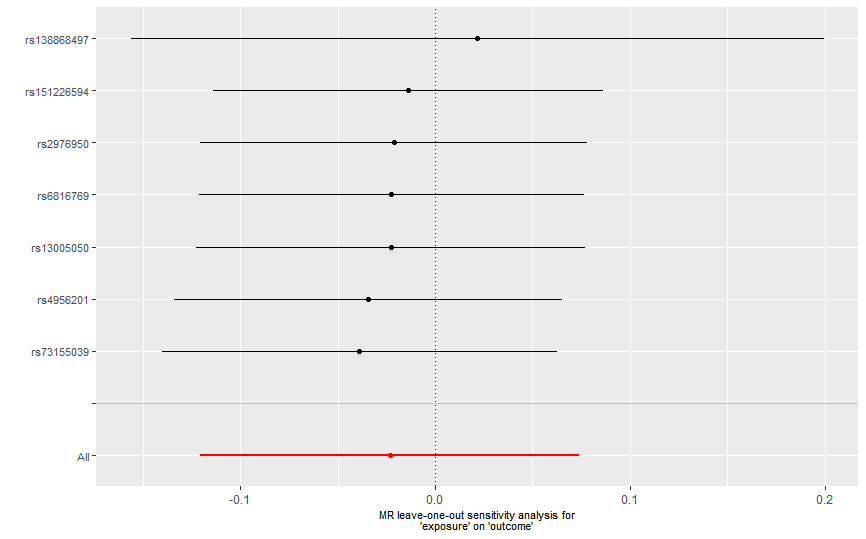


(a)


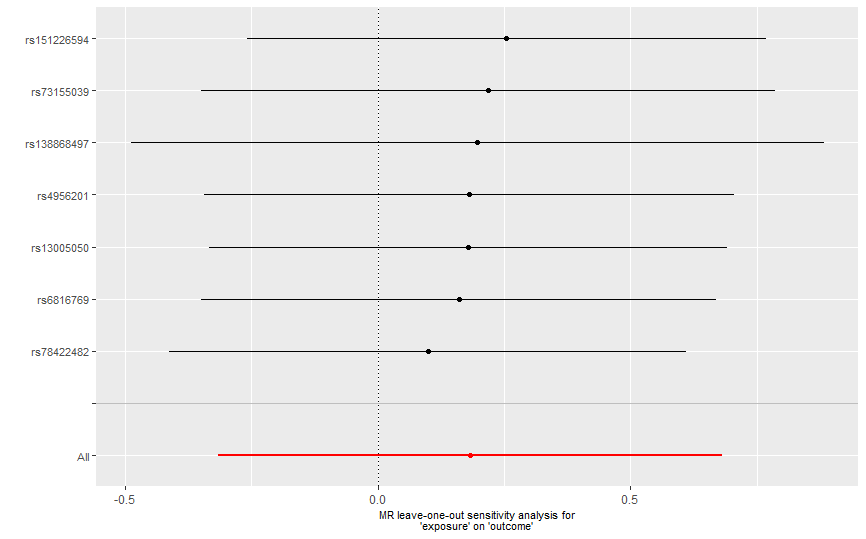


(b)


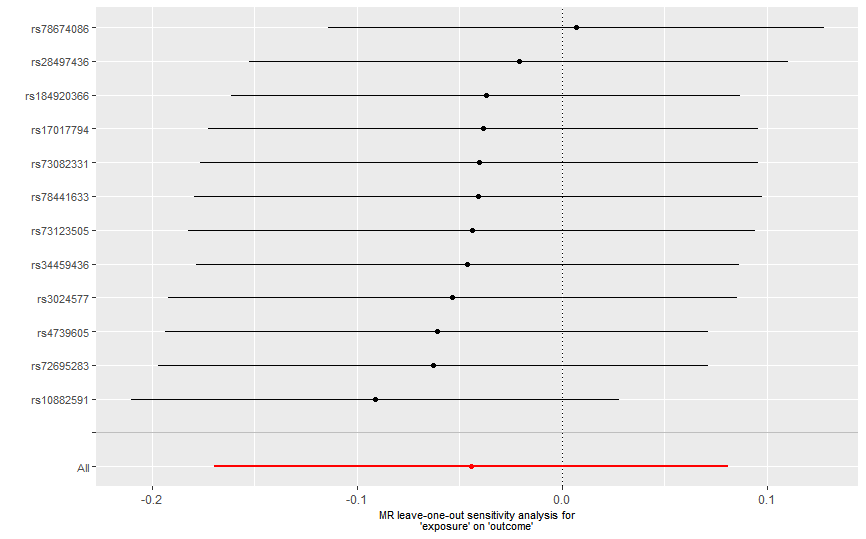


(c)
